# Feasibility and Impact of Integrating an Artificial Intelligence–Based Diagnosis Aid for Autism Into the Extension for Community Health Outcomes Autism Primary Care Model: Protocol for a Prospective Observational Study

**DOI:** 10.2196/37576

**Published:** 2022-07-19

**Authors:** Kristin Sohl, Rachel Kilian, Alicia Brewer Curran, Melissa Mahurin, Valeria Nanclares-Nogués, Stuart Liu-Mayo, Carmela Salomon, Jennifer Shannon, Sharief Taraman

**Affiliations:** 1 ECHO Autism Communities University of Missouri School of Medicine Columbia, MO United States; 2 SSI Strategy Parsippany, NJ United States; 3 Cognoa, Inc Palo Alto, CA United States; 4 Children’s Health of Orange County Orange, CA United States; 5 Department of Pediatrics School of Medicine University of California, Irvine Irvine, CA United States; 6 Dale E and Sarah Ann Fowler School of Engineering Chapman University Orange, CA United States

**Keywords:** autism spectrum disorder, diagnosis, artificial intelligence, primary care, machine learning, Software as a Medical Device, mobile phone

## Abstract

**Background:**

The Extension for Community Health Outcomes (ECHO) Autism Program trains clinicians to screen, diagnose, and care for children with autism spectrum disorder (ASD) in primary care settings. This study will assess the feasibility and impact of integrating an artificial intelligence (AI)–based ASD diagnosis aid (the *device*) into the existing ECHO Autism Screening Tool for Autism in Toddlers and Young Children (STAT) diagnosis model. The prescription-only Software as a Medical Device, designed for use in children aged 18 to 72 months at risk for developmental delay, produces ASD diagnostic recommendations after analyzing behavioral features from 3 distinct inputs: a caregiver questionnaire, 2 short home videos analyzed by trained video analysts, and a health care provider questionnaire. The device is not a stand-alone diagnostic and should be used in conjunction with clinical judgment.

**Objective:**

This study aims to assess the feasibility and impact of integrating an AI-based ASD diagnosis aid into the ECHO Autism STAT diagnosis model. The time from initial ECHO Autism clinician concern to ASD diagnosis is the primary end point. Secondary end points include the time from initial caregiver concern to ASD diagnosis, time from diagnosis to treatment initiation, and clinician and caregiver experience of device use as part of the ASD diagnostic journey.

**Methods:**

Research participants for this prospective observational study will be patients suspected of having ASD (aged 18-72 months) and their caregivers and up to 15 trained ECHO Autism clinicians recruited by the ECHO Autism Communities research team from across rural and suburban areas of the United States. Clinicians will provide routine clinical care and conduct best practice ECHO Autism diagnostic evaluations in addition to prescribing the device. Outcome data will be collected via a combination of electronic questionnaires, reviews of standard clinical care records, and analysis of device outputs. The expected study duration is no more than 12 months. The study was approved by the institutional review board of the University of Missouri-Columbia (institutional review board–assigned project number 2075722).

**Results:**

Participant recruitment began in April 2022. As of June 2022, a total of 41 participants have been enrolled.

**Conclusions:**

This prospective observational study will be the first to evaluate the use of a novel AI-based ASD diagnosis aid as part of a real-world primary care diagnostic pathway. If device integration into primary care proves feasible and efficacious, prolonged delays between the first ASD concern and eventual diagnosis may be reduced. Streamlining primary care ASD diagnosis could potentially reduce the strain on specialty services and allow a greater proportion of children to commence early intervention during a critical neurodevelopmental window.

**Trial Registration:**

ClinicalTrials.gov NCT05223374; https://clinicaltrials.gov/ct2/show/NCT05223374

**International Registered Report Identifier (IRRID):**

PRR1-10.2196/37576

## Introduction

### Background

Autism spectrum disorder (ASD), a neurodevelopmental disorder impacting both communication and behavior, affects approximately 1 in every 44 children in the United States [1]. Targeted early intervention during a critical neurodevelopmental window is recommended to maximize long-term cognitive gains [2,3], adaptive behaviors [4-6], social and emotional functioning [7,8], and verbal fluency [8,9]. ASD evaluations have traditionally been managed in specialist settings; however, a rapid rise in prevalence rates over the past half century [10] has outpaced specialist capacity and led to prolonged wait times for diagnostic evaluations [10-13]. Currently, families in the United States may wait as long as 18 months between initial screening by their primary care clinician and diagnosis by a specialist [11].

Rising ASD prevalence rates, combined with specialist shortages, have contributed to significant diagnostic delays in the United States. Although ASD can be reliably diagnosed at as early as 18 months, the mean age of ASD diagnosis has remained >4 years since the Centers for Disease Control and Prevention began tracking prevalence rates [1,14-16]. The average 3 years delay from first parental concern to eventual diagnosis recorded in the literature is even longer for demographic groups that include girls, children of color, and children who are rural residing or of lower socioeconomic status [17-20]. An estimated 27% of children with ASD in the United States remain undiagnosed at the age of 8 years [20].

Given the importance of early diagnosis and intervention, the American Academy of Pediatrics has recommended that all children be screened in primary care at 18 and 24 months, in addition to whenever caregivers express concern [12]. When they feel confident, upon a failed screen, primary care clinicians are encouraged to make the ASD diagnosis themselves using the Diagnostic and Statistical Manual of Mental Disorders–fifth edition (DSM-5) criteria [12]. Alternatively, they can refer to specialists for further assessment. However, studies suggest that clinicians do not consistently screen or refer to these recommendations [21]. Currently, <1% of all ASD diagnoses are made by primary care clinicians in the United States [21,22].

The low rates of ASD diagnosis in primary care settings may be explained by several factors. Clinician–identified barriers include a lack of ASD-specific training and low self-efficacy in making the diagnosis, as well as time constraints that limit their ability to comprehensively evaluate, review results, and discuss treatment plans with families in primary care [23-26]. Complex presentations in children with ASD, including multiple overlapping comorbidities and a diverse spectrum of symptoms, can also confound the diagnosis [27]. Although diagnostic tools have been developed to support accurate evaluations [28], *gold standard* ASD diagnostic instruments with the highest validity [28-30] are typically poorly suited to use in primary care. This is because they are time intensive and typically require in-person delivery in a clinical facility and specialist training for reliable administration. Even when clinicians receive specialist training, primary care reimbursement rates may be insufficient to justify the effort [31].

### Device Development

**Figure 1:**
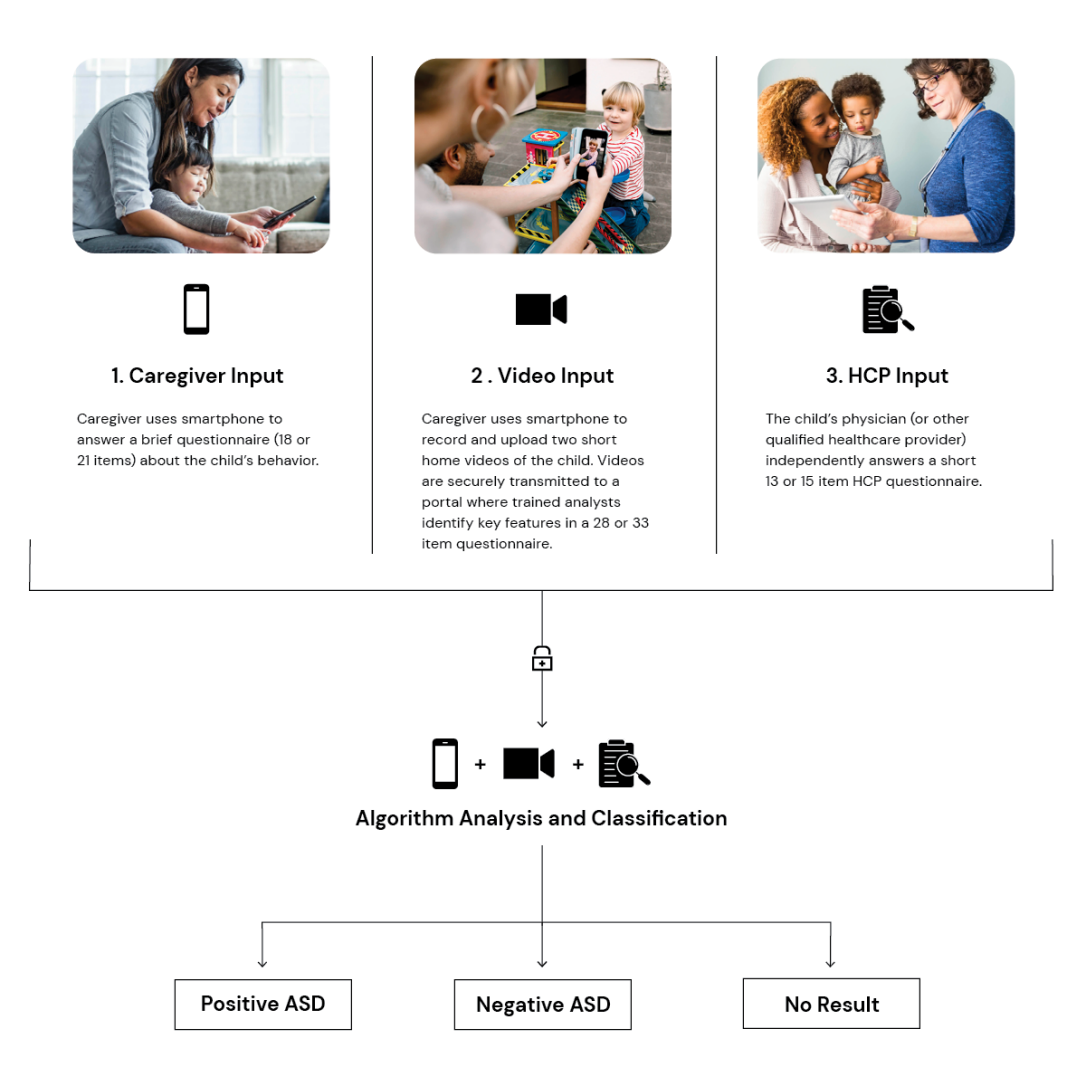
Overview of device use. ASD: autism spectrum disorder; HCP: health care provider.

Diagnosis aids tailored specifically for use in primary care are urgently needed to streamline ASD evaluations and enhance primary care diagnostic capacity. In response to this need, an artificial intelligence (AI)–based Software as a Medical Device [32] was developed to support health care providers in diagnosing or ruling out ASD in children aged 18 to 72 months with concerns for developmental delay. The device is indicated for use by health care providers who have prescriptive authority in their state of practice. For the purposes of this study the device will be used exclusively by primary care Extension for Community Health Outcomes (ECHO) Autism clinicians. The device was developed using patient record data from thousands of children with diverse conditions, presentations, and comorbidities who were either diagnosed with ASD or confirmed not to have ASD based on standardized diagnostic tools [29,30] and representing both sexes across the supported age range. The underlying device algorithm was iteratively improved, supplemented with ASD expert input, and prospectively validated for over 7 years [33-38]. Key steps involved in device use are illustrated in Figure 1.

The device integrates 3 independent inputs within its gradient-boosted decision tree algorithm: a brief caregiver questionnaire (completed via a mobile app), a video analyst questionnaire based on the analysis of 2 short home videos uploaded by the caregiver (completed in the video analyst portal), and a health care provider questionnaire (completed in the health care provider portal). The device evaluates inputs based on the predictive features that are most indicative of ASD. If the information provided is sufficiently granular, the device will provide an *ASD Positive* or *ASD Negative* output. When a highly predictive determination cannot be made, the device abstains from providing a result (*No Result* output). Following strong pivotal trial results [39], the device received Food and Drug Administration (FDA) marketing authorization on June 2, 2021 [40]. In a prospective, double-blinded multisite pivotal trial, the device was found to facilitate timely and accurate ASD diagnostic evaluation in nearly a third of the study cohort while minimizing false negatives to maintain clinical safety [39]. However, real-world evidence is needed to learn more about the utility and impact of the device in everyday US primary care settings.

### The ECHO Autism Primary Care Model

ECHO Autism primary care [41,42] is a virtual learning program in which primary care clinicians receive guidance from ASD experts regarding best practices for ASD screening, identification, and management of medical and psychiatric comorbidities. The diagnostic component of the ECHO Autism primary care model involves training clinicians to conduct diagnostic assessments for ASD using the Screening Tool for Autism in Toddlers and Young Children (STAT), a level 2 ASD screener used as a diagnostic observational measure. The ECHO Autism STAT model provides training and mentorship to equip clinicians to diagnose ASD in cases that are clear and unambiguous and appropriately identify and refer complex cases for further assessment [43]. A 12-month pilot study with 18 participating clinicians was conducted using the ECHO Autism STAT model [43]. In this study, participating clinicians presented the results of their assessment to the ECHO Autism expert hub team for specialist input before guiding the diagnostic pathway to include either a diagnosis of ASD or a referral for further specialist evaluation. This pilot study showed that 73% of the participants reported acceptance of referrals for ASD diagnostic evaluations following program completion, and 80% reported an increase in the number of children with ASD in their caseloads. In addition, the pilot demonstrated a 2- to 6-month decrease in time to access services when compared with standard-of-care diagnostic pathways [41]*.*

### Study Objectives

The objective of this study is to assess the feasibility and impact of integrating the device into the existing primary care diagnostic pathway, the *ECHO Autism STAT model*.

## Methods

### Ethics Approval

The study was approved by the institutional review board of the University of Missouri-Columbia (institutional review board–assigned project number 2075722) and registered with ClinicalTrials.gov (NCT05223374).

### Study Design

This prospective observational study was designed to obtain real-world evidence pertaining to the feasibility and impact of integrating the device into a primary care ASD diagnostic pathway. Demographic, clinical, and qualitative user experience data will be obtained from eligible participants. All decisions regarding patient care will be determined by the ECHO Autism clinicians, as the study is noninterventional. All clinical outcomes will be assessed by the ECHO Autism clinician, as in routine clinical practice.

### Study Population

#### ECHO Autism Clinicians

##### Inclusion Criteria

Clinicians eligible for study participation will be practicing primary care physicians and nurse practitioners who are able to evaluate patients for ASD as part of their scope of practice. All clinicians must have received training in the ECHO Autism Communities diagnostic model from the University of Missouri ECHO Autism team before study enrollment.

##### Exclusion Criteria

Clinicians who are unable to evaluate patients with suspected ASD or who have not completed the ECHO Autism diagnostic training will be excluded from study enrollment.

##### ECHO Autism Program Recruitment

Starting in 2016, practicing primary care clinicians located in geographically diverse areas across Missouri were invited to participate in ECHO Autism STAT. Participants were identified through word of mouth, professional association listservs, and strategic recruitment from underserved locations. This program was a continuation of the existing ECHO Autism primary care program that started in 2015. A 2-day training was added focused on use of a direct behavior observation tool (STAT) and comprehensive diagnostic interviewing. The ECHO Autism STAT participants completed in-person training, regularly participated in ECHO Autism telementoring sessions, and achieved reliability with the certified trainer on the STAT observation. After completion of the training elements, these participants were eligible to bring diagnostic cases for discussion during the tele–ECHO Autism sessions. Only clinicians completing all elements of the ECHO Autism diagnostic training are eligible for participation in this study. Eligible clinician participants will be informed of the planned study, offered 2 sessions to ask questions about study participation, and consented through usual research procedures.

#### Patients and Caregivers

##### Inclusion and Exclusion Criteria

At the time of screening, patients must meet all of the inclusion criteria (Textbox 1) to be eligible for study enrollment. Patients who meet any of the exclusion criteria (Textbox 1) will not be eligible for enrollment in the study.

**Inclusion and exclusion criteria.**

**Inclusion criteria**

- Must be aged 18 to 72 months
- Must have developmental delay concerns expressed by caregivers, clinicians, or other community-based professionals
- The participating caregiver must speak English proficiently in the opinion of the clinician.
- Must be able to read, understand, sign, and date the informed consent form
- Must have a smartphone using either an iOS or Android operating system (as of June 2022, iOS 14 or 15 and Android 11 or 12, excluding Android Go, are supported; future updates are planned to ensure ongoing compatibility with new operating system versions as they are released)
- Must be willing to have the child videotaped as part of the device input
- Must meet device labeling requirements

**Exclusion criteria**

- Participants with a prior diagnosis of autism spectrum disorder rendered by a clinician
- Participants having any other medical, behavioral, or developmental condition that in the opinion of the clinician, may confound study data or assessments
- Participants whose age on the date of enrollment is outside the target age range
- Participants who have, to the best of their knowledge, been previously enrolled in any clinical study or survey involving the device or who have a caregiver who has been enrolled in any such study
- Participants whose medical records have been included in any internal device machine learning training or validation sets

##### Patient Recruitment

Participants will be recruited from the population of patients that the ECHO Autism clinician would see as part of their usual enhanced primary care practice. As part of routine practice, ECHO Autism clinicians receive referrals from external primary care clinicians, and when the referrals are received, families will be informed of the opportunity to participate in the study if deemed eligible. Participating clinicians or their designees will inform caregivers of children presenting with a concern for developmental delay about the study. Additional recruitment strategies such as social media, flyers, and email communication with community clinicians may be used as needed to reach recruitment goals.

### Study Flow

#### Overview

**Figure 2:**
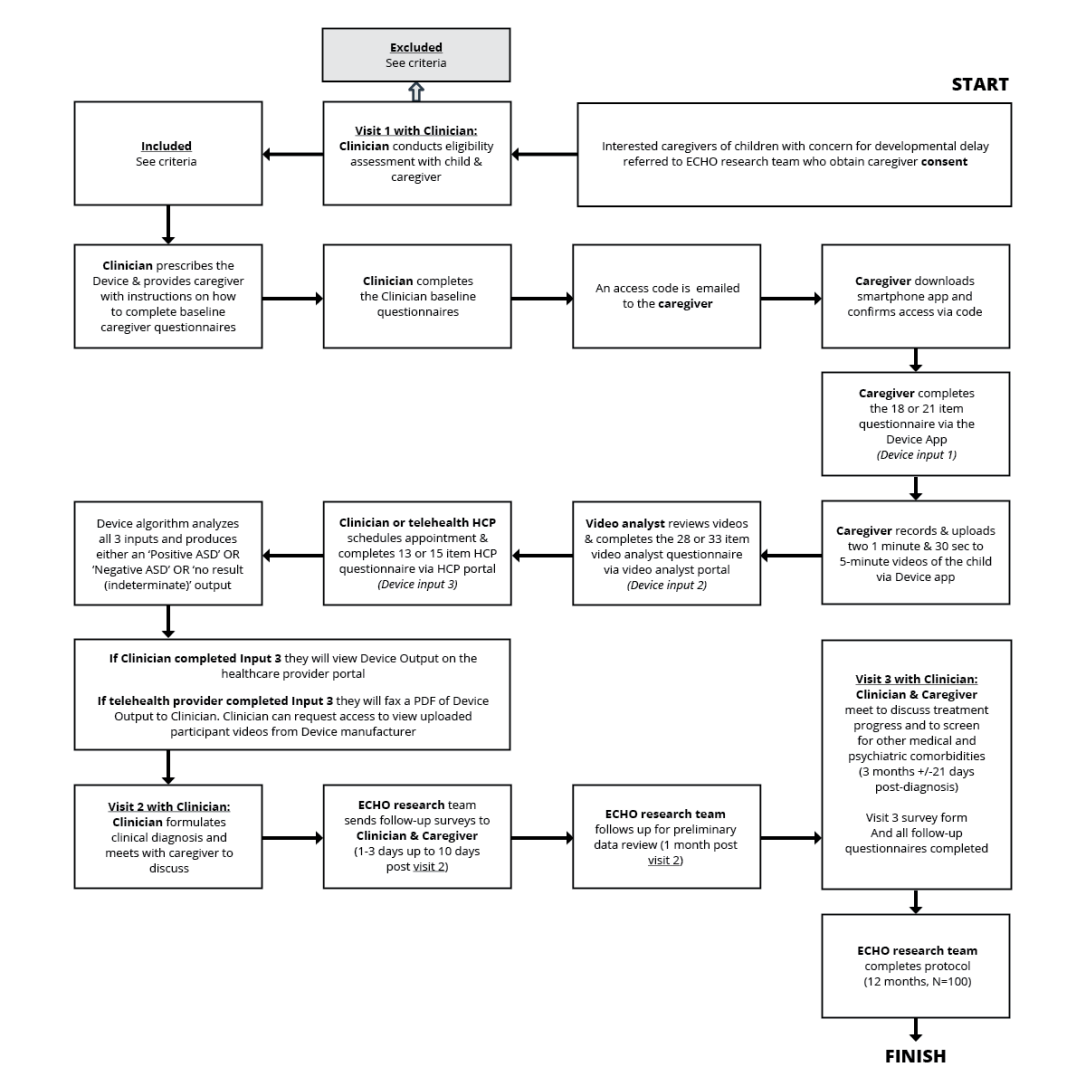
Study flow. ASD: autism spectrum disorder; ECHO: Extension for Community Health Outcomes; HCP: health care provider.

This study is being conducted by the University of Missouri ECHO Autism Communities Research Team. Eligible caregivers of patients with a concern for developmental delay and ECHO Autism clinicians will complete the informed consent process before entering the study. Once enrolled, ECHO Autism clinicians will provide routine clinical care and conduct best practice ECHO Autism diagnostic evaluations with the addition of device prescriptions for patients suspected of having ASD. Patients will be followed up with routine clinical care on the schedule shown in Figure 2. Data will be collected at the end of the 3-month follow-up period. The clinician will complete a visit form for any interim visits that occur between visit 1 and the 3-month follow-up appointment.

The study flow is summarized in Figure 2 and described in detail in the following sections.

#### Visit 1: Eligibility Determination

An appointment is scheduled for a participant (patient) with concern for developmental delay and their caregiver. During the first clinic visit, the ECHO Autism clinician will observe the participant and conduct a DSM-5 interview to determine whether the participant requires further ASD evaluation.

#### Baseline Data Collection

The ECHO Autism clinician will connect the caregiver of any potential participant requiring further evaluation for ASD to the University of Missouri ECHO Autism Communities research team. Before visit 1, the research team will obtain consent from the family. The research team will provide instructions on how to complete the electronic study baseline caregiver questionnaires and download the device’s caregiver app. The research team will also send electronic baseline ECHO Autism clinician questionnaires to the clinician to complete. The clinician will prescribe the device.

#### Device Completion

Once the device is prescribed by the clinician, the study sponsor will release the access code to the study team, who will then email it to the caregiver. The caregiver will download the mobile app to their smartphone and access the app using the code. The caregiver will then complete an 18- or 21-item age-dependent caregiver questionnaire (*device input 1*) and record and upload two 1.5-minute to 5-minute videos of the child on the mobile app. The uploaded videos will be remotely reviewed and scored on a 28- or 33-item video analyst questionnaire (*device input 2*) by a trained video analyst via the video analyst portal*.* The ECHO Autism clinician then has the option to *either* complete the health care provider questionnaire, which is a 13- or 15-item age-dependent questionnaire (*device input 3*), themselves or schedule the participant for a telehealth provider appointment, during which the questionnaire will be completed by the telehealth provider. The device algorithm then analyzes all 3 inputs and produces either a *positive ASD*, *negative ASD*, or *indeterminate/no result* output for the clinician to review.

#### Visit 2: Diagnostic Results Meeting

After the device output is received, the clinician will synthesize their diagnostic impressions (ie, in-person observation, DSM-5 interview, and questionnaires) and the device output to determine whether the participant meets the criteria for a diagnosis of ASD. The clinician may also request access to view the participant’s videos when making a diagnosis. In cases where the clinician’s judgment conflicts with the device output, the diagnostic determination of the clinician (who remains the decision-maker in the diagnostic process) takes precedence. This is consistent with the device’s FDA market authorization status as a diagnosis aid intended to support health care providers in diagnosing ASD rather than as a stand-alone diagnostic device.

For participants whom the clinician determines as meeting the criteria of ASD, the clinician will deliver the diagnosis to the caregiver during visit 2. In addition, the clinician will schedule a 3-month follow-up visit. The research team will then send electronic postdiagnostic questionnaires to the caregiver and the clinician to be completed 1 to 3 (up to 10) days after visit 2.

#### Visit 3: Treatment Progress Meeting

The clinician will follow-up with the caregiver for 3 months (+21 or –21 days) after the participant receives an ASD diagnosis to discuss treatment progress and screen for any other medical and psychiatric comorbidities. The research team will send electronic postdiagnostic questionnaires to the caregiver and clinician to complete 1 to 3 (up to 10) days after this follow-up visit.

#### Interim Visit Forms

Clinicians will complete interim visit forms for any additional visits that may occur between study enrollment and the scheduled final 3-month follow-up visit.

#### Long-term Follow-up

Participants and their caregivers may be considered for enrollment if they choose to consent to an extension study to continue data collection related to long-term outcomes.

### Number of Sites Enrolling Participants

Up to 15 University of Missouri ECHO Autism clinicians in both rural and suburban areas across the United States will be recruited.

### Study Duration

Study enrollment is expected to take approximately 12 months. Patient participation will occur from the time the ECHO Autism clinician identifies a concern for ASD through +21 or −21 days of the 3-month follow-up visit.

### Potential Risks and Benefits of Participation

There are no physical risks associated with participation in the study. Caregivers may feel some psychological discomfort when answering questions about their children’s behaviors. Children might also feel some psychological discomfort when being filmed for the video analysis. Videos will be used to evaluate participants’ behaviors as part of the diagnostic process. The videos will be captured within the mobile app, stored on Amazon’s secure cloud service (Amazon S3), and analyzed by the device manufacturer through a secure web portal. By agreeing to participate in the study, participants will grant and release all rights, titles, and interests in the videos to the device manufacturer. Another potential risk is the possible release of sensitive medical, behavioral, or educational information. To mitigate the risk of releasing sensitive personal information, the device manufacturer will adhere to confidentiality protocols aligned with the US Health Insurance Portability and Accountability Act and FDA 21 Code of Federal Regulations Part 11, as well as other federal, national, and local laws and regulations, to protect the privacy of personal information. All content within the mobile app will be stored on Amazon S3 and analyzed by the device manufacturer through a secure web portal. When data are published, they will be anonymized in a manner that prevents the identification of specific caregivers or participants. To the best of the manufacturer’s knowledge, there were no data or security breaches in any prior studies involving the device.

A potential benefit of the study is that device use could support ECHO Autism clinicians in identifying participants with ASD earlier than they would otherwise have been identified, thus enabling earlier therapy, which is known to positively affect long-term outcomes.

### Participant Withdrawal or Termination

Participation is completely voluntary. Caregivers may withdraw consent for study participation at any time by informing the principal investigator or the study staff. The principal investigator may also withdraw a participant from the study if determined that further participation would not be in the participant’s best interest. Participants may be discontinued by the study staff if determined unable to fully meet the requirements of participation, including the completion of study visits and necessary assessments. The reasons for discontinuation will be recorded for all participants. If lost to follow-up, the research team will attempt to contact the caregiver by telephone, followed by registered mail, to establish the reason for withdrawal as completely as possible.

### Study Objectives

#### Primary Objective

The primary objective is to assess the time from the initial concern by the ECHO Autism clinician to diagnosis when using the device as part of the diagnostic pathway.

#### Secondary Objectives

Secondary objectives include the collection and analysis of data from both the ECHO Autism clinician and caregiver participants to learn about the information described in Textbox 2.

**Data collected for secondary objectives.**

**Data collected for secondary objectives**

- Patient outcomes correlating with time to initiating therapy following a positive autism spectrum disorder (ASD) diagnosis
- Distance from patient home to nearest ASD specialist center
- Level of clinician self-efficacy in using the device as part of the evaluation and diagnosis of children with suspected ASD
- Qualitative data on the following: caregiver satisfaction with the diagnostic process; time from caregiver concern to diagnosis; clinician certainty rating for diagnostic determination; clinician satisfaction with using the device as part of the Extension for Community Health Outcomes (ECHO) Autism diagnostic process; caregiver perception of the device with validated questionnaire; determination of whether the use of the device enhances the efficiency and feasibility of the ECHO Autism Communities diagnostic process; likelihood to recommend (clinician and caregiver); efficiency and feasibility of ECHO Autism Community–based diagnostic process, as measured by self-efficacy, certainty ratings, perceived barriers, and practice patterns questionnaires; percentage of positive outcomes correlating with shorter durations between diagnosis and treatment; percentage of caregivers who feel they received a high-quality evaluation; percentage of clinicians with increased self-efficacy in using the device as part of the diagnostic process; percentage of clinicians finding the device helpful in the diagnostic process; percentage of positive device perceptions from caregiver; barriers to app use for caregivers; clinician-perceived barriers to practice implementation of device
- Assessment of insurance coverage or reimbursement (although insurance will not be billed for this study)
- Amount of time required for clinicians to make a diagnosis
- Percentage of clinician participants reporting a positive change in practice flow because of using the device as part of their diagnostic process
- Percentage of patients for whom the device can provide a determinate result (ASD positive or negative)
- Summary of interventional treatments recommended by the clinician following diagnosis
- Changes in clinician clinical practice flow
- Change in quality of life measures
- Time associated with device use

### Data Collection Instruments and Schedule of Assessments

#### Clinical History

Details of participants’ clinical and medical histories will be reviewed and captured, including the description and timing of the initial concern for ASD.

#### Diagnostic Procedures

Diagnostic procedures will be completed according to the ECHO Autism Communities standard of care, as determined by the treating clinician in routine clinical practice. Details of any such procedure will be captured throughout the study, including the follow-up period after a participant has received diagnostic results.

#### Therapeutic Interventions

Any new therapeutic interventions recommended by the ECHO Autism clinician will be captured at time points 1, 2, and 3 and for any interim visit. Once a recommendation has been made, data will be collected during subsequent visits to indicate patient compliance with the recommendations.

All data collection instruments are listed and described in Textbox 3.

**Data collection instruments.**

**Caregiver questionnaire (device input 1)**

- This is not a stand-alone assessment. The caregiver questionnaire will be completed within the mobile caregiver app upon receipt of the access code following completion of the informed consent form. This is an 18- or 21-item questionnaire that collects details related to the caregiver’s observations of the participant’s developmental behaviors.

**Video analyst questionnaire (device input 2)**

- This is not a stand-alone assessment. Video analysts will answer a 28- or 33-item age-dependent questionnaire in the video analyst portal after viewing the 2 short video submissions uploaded by the caregiver. Video analysts will evaluate the observed behaviors by answering a series of multiple-choice questions evaluating phenotypic features of autism spectrum disorder (ASD) such as nonverbal and verbal communication, social interaction, unusual sensory interests or reactions, stereotypic or repetitive motor movements, use of objects, or speech on the combinative videos.

**Health care provider questionnaire (device input 3)**

- This is not a stand-alone assessment. The clinician or telehealth provider will schedule a visit with the caregiver and participant (which can be remote). During this visit, the participant must be available for observation. The clinician or telehealth vendor will complete the 13- or 15-item questionnaire within the health care provider portal. The questionnaire addresses aspects of the child’s behavior and development.

**Device output**

- Device output is produced by the device summarizing findings after algorithmic analysis of the 3 device inputs and contains one of 3 possible results: positive ASD, negative ASD, or no result. It is to be used by the clinician as part of the Extension for Community Health Outcomes (ECHO) Autism Community–based diagnostic process.

**Measure of processes of care**

- This measure assesses the family’s perceptions of the care they and their child receive within a particular setting. It is a means to assess the family-centered behaviors of clinicians. This is a validated measure that has been adapted for this study.

**ECHO Autism caregiver presurvey**

- This survey collects initial demographic information regarding each family and assesses the family’s initial perception of the ECHO Autism clinician as they begin the diagnostic process.

**ECHO Autism caregiver postdiagnostic survey**

- This survey assesses the caregiver’s satisfaction with the services they received.

**ECHO Autism clinician preproject survey**

- This survey measures the ECHO Autism clinician’s self-efficacy, perceived barriers, and satisfaction with the current ECHO Autism standard-of-care procedure.

**ECHO Autism clinician postproject survey**

- This survey measures the self-efficacy, perceived barriers, and satisfaction with the use of the device as part of the ECHO Autism standard-of-care procedure.

**ECHO Autism 3-month follow-up questionnaires**

- The purpose of these questionnaires is to address the data points required for the study’s secondary and exploratory objectives.

**ECHO Autism interim visit form**

- The purpose of the form is to summarize any visit from a research participant occurring between the time of concern and the 3-month follow-up visit.

### Statistical Analyses

#### Statistical and Analytical Plans

Descriptive statistics will be used to summarize the data. Continuous variables will be summarized as n, mean, SD, median, minimum, and maximum values. Categorical variables will be summarized as participant counts and related percentages.

#### General Approach

All available data will be included in data listings and tabulations. Data will be summarized by participant counts and related percentages. In general, categorical data will be presented using counts and percentages, whereas continuous variables will be presented using mean, SD, median, minimum, and maximum values. Counts of observed and missing data will also be reported.

#### Safety Analysis

Device- and procedure-related adverse effects will be tabulated and analyzed.

#### Sample Size

This is a prospective study with up to 100 patient participants determined eligible for an evaluation for ASD and up to 15 ECHO Autism clinicians who conduct assessments.

## Results

Participant recruitment began in April 2022. As of June 2022, a total of 41 participants have been enrolled in the study. Data collection is anticipated to continue through 2022, with the data analysis and publication of findings being planned for 2023.

## Discussion

### Principal Findings

To our knowledge, this collaborative observational study will be the first to evaluate the use of a novel AI-based ASD diagnosis aid as part of a real-world primary care diagnostic pathway. It is anticipated that this study will provide valuable feasibility data regarding device integration into a primary care workflow. The study results may help clarify the length of time between initial clinician concern and diagnosis when an AI-based device is used as part of the evaluation process. The results may also provide insights into device performance outside of a research setting, including the percentage of primary care participants for whom it is able to provide a determinate ASD positive or negative result, and concordance between device output and clinical diagnostic determination. Qualitative user experience measures captured as part of the study are anticipated to shed light on the acceptability of the device to both families and clinicians and may help drive future product improvements.

### Comparison With Previous Literature

Although to date, no previous AI-based ASD diagnosis aid primary care–focused studies have been conducted, there is literature supporting the feasibility of diagnosing and managing ASD in primary care settings. For example, a recent systematic review focused on training clinicians to diagnose and manage ASD in primary care [44] found that with appropriate training and support, primary care clinicians can render diagnostic decisions that closely align with specialty team assessments. The reviewed studies also reported reduced wait times for diagnosis when a proportion of evaluations were moved to the primary care setting.

### Strengths and Limitations

Despite well-documented bottlenecks in ASD diagnosis and treatment initiation in the United States [11,45], to date, very few diagnostic tools have been designed to enhance primary care diagnostic capacity. The integration of the device into the ECHO Autism primary care diagnostic pathway in this study offers several potential benefits over existing tools [28-30]. As it was designed with primary care use in mind, the device is more time-efficient than existing instruments and does not require specialist training to administer. For example, in the pivotal trial, the total time burden associated with device use was <30 minutes (median time for caregiver questionnaire completion was 4 minutes and 56 seconds; median time for video analyst scoring from the time video review began to submission of scores was 10 minutes and 54 seconds; self-reported time for health care providers to complete their questionnaire was approximately 10 minutes) [39].

The device is also amenable to remote administration. Caregivers will complete their questionnaires and upload videos remotely using an app on their mobile phones. Video analysts will receive inputs and upload questionnaire outputs remotely through an analyst portal. Pivotal trial data showed no evidence of performance degradation in cases where health care providers completed their questionnaires remotely versus in person. Remote administration may support diagnostic access for families residing in rural and remote locations, as well as families without access to transportation. Remote administration is also useful during public health emergencies such as the COVID-19 pandemic.

Compared with other ASD assessment instruments where the child is observed in a clinical setting, the device leverages home videos that provide naturalistic data about the child’s behavior outside of the clinical setting. These data may help to overcome some of the challenges clinicians and caregivers have described capturing typical child behavior within time-limited clinical encounters [46]. For example, in the clinical setting, the child may become overly reactive to changes in the environment or may not display the behaviors of concern they exhibit at home. Without a picture of the child’s typical behavior, it can be challenging for the clinician to make a confident diagnostic assessment.

The device was also trained on a large and diverse data set in terms of race and ethnicity, socioeconomic status, and gender. Although the pivotal trial was not powered for statistical inference on these covariates, our initial analysis indicated no evidence of inconsistent device performance across these variables [39]. This finding is encouraging and points to the potential of the device to address some of the well-documented disparities in current diagnostic approaches whereby girls and African American and Hispanic children are misdiagnosed more often, as well as diagnosed at a later age, on average, than other groups [47,48].

The device itself and the overall study design have several constraints and limitations. Device use requires access to a smartphone and wireless internet, which not all low-income American families have [49]. In addition, the device is only available in English. Thus, many culturally and linguistically diverse potential participants will be excluded from the study. Selection bias may also be a concern in this study. For example, parents with a higher number of concerns about their child’s development may be more motivated to enroll in the study and complete all device inputs than parents with milder concerns. Attrition is another concern in this study as participation involves multiple steps and engagement over a 3-month period. Given the multiple steps involved, it is possible that study completers may end up representing a more motivated cohort than study leavers. The clinicians who will be recruited into the trial may also represent a biased sample, as by enrolling in ECHO training, they have already demonstrated an interest in enhancing their knowledge of developmental disabilities. In addition, owing to the ECHO Autism curriculum training they receive, clinicians may have more experience and background in ASD diagnosis than other primary care clinicians. In other settings, clinicians with less interest in ASD may be less motivated or confident in using the device and may be less willing to diagnose in the primary care setting.

### Future Directions

The study follows participants for a total of 3 months. Additional follow-up studies are recommended to assess the stability of device performance over a longer period. For the purposes of this study, the sponsors will cover the device cost and associated clinician fees. However, if the device were to become a standard tool in primary care settings, it is unknown to what extent insurance companies would accept and reimburse these costs. Questions also remain regarding whether and how families with no health insurance can access the device. We recommend that future device studies be conducted in populations who have not completed the ECHO Autism diagnostic training program. Such research could help answer important questions about the feasibility of device use in primary care clinician populations with less ASD-specific knowledge and training.

### Conclusions

If device integration into primary care practice proves feasible and efficacious, it may support clinicians in diagnosing more children in the primary care setting and reducing current delays between the first ASD concern and eventual diagnosis. Streamlining primary care ASD diagnosis could potentially reduce strain on specialist services by allowing for the diagnosis of less ambiguous cases in primary care and quicker referral of complex cases to relevant specialist teams. Ultimately, we hope that decreasing the time to diagnosis and age of diagnosis through enhanced primary care capacity will allow a greater proportion of children to commence early intervention during a critical neurodevelopmental window.

## Abbreviations

AI: artificial intelligence
ASD: autism spectrum disorder
DSM-5: Diagnostic and Statistical Manual of Mental Disorders–fifth edition
ECHO: Extension for Community Health Outcomes
FDA: Food and Drug Administration
STAT: Screening Tool for Autism in Toddlers and Young Children
